# Is mid-life social participation associated with cognitive function at age 50? Results from the British National Child Development Study (NCDS)

**DOI:** 10.1186/s40359-016-0164-x

**Published:** 2016-12-02

**Authors:** Ann Bowling, Jitka Pikhartova, Brian Dodgeon

**Affiliations:** 1 Faculty of Health Sciences, University of Southampton, Highfield Campus, Southampton, SO171BJ UK; 2 Brunel University London, College of Health and Life Sciences, Department of Clinical Sciences, London, UK; 3 Centre for Longitudinal Studies, UCL Institute of Education, 20 Bedford Way, London, WC1H 0AL20 UK

**Keywords:** Cognitive function, Civic engagement, Predictor, Longitudinal, Cohort

## Abstract

**Background:**

Some studies have indicated that social engagement is associated with better cognitive outcomes. This study aimed to investigate associations between life-course social engagement (civic participation) and cognitive status at age 50, adjusting for social networks and support, behavioural, health, social and socio-economic characteristics.

**Methods:**

The vehicle for the study was the National Child Development Study (1958 Birth Cohort Study), which is a general population sample in England, Scotland and Wales (9119: 4497 men and 4622 women) participating in nationally representative, prospective birth cohort surveys. The primary outcome variable was cognitive status at age 50, measured by memory test (immediate and delayed word recall test) and executive functioning test (word fluency and letter cancelation tests). The influence of hypothesised predictor variables was analysed using linear multiple regression analysis.

**Results:**

Cognitive ability at age 11 (β = 0.19;95% CI = 0.17 to 0.21), participation in civic activities at ages 33 (0.12; 0.02 to 0.22) and 50 (0.13; 0.07 to 0.20), frequent engagement in physical activity (sport) (β from 0.15 to 0.18), achieving higher level qualifications (β from 0.23 to 1.08), and female gender (β = 0.49;95% CI = 0.38 to 0.60) were positively, significantly and independently associated with cognitive status at age 50. Having low socio-economic status at ages 11 (β from -0.22 to -0.27) and 42 (β from -0.28 to -0.38), and manifesting worse mental well-being at age 42 (β = -0.18; 95% CI = -0.33 to -0.02) were inversely associated with cognitive status at age 50. The proportion of explained variance in the multiple regression model (18%), while modest, is impressive given the multi-faceted causal nature of cognitive status.

**Conclusions:**

The results indicate that modest associations between adult social engagement and cognitive function at age 50 persist after adjusting for covariates which included health, socio-economic status and gender, supporting theories of neuroplasticity. In addition to the continuing emphasis on physical activity, the encouragement of civic participation, at least as early as mid-life, should be a targeted policy to potentially promote and protect cognitive function in later mid-life.

**Electronic supplementary material:**

The online version of this article (doi:10.1186/s40359-016-0164-x) contains supplementary material, which is available to authorized users.

## Background

Decline in cognitive and physical functioning is likely to reflect interactions between a person’s genes, biology, socio-economic and environmental circumstances, behaviour, socio-psychological and physical reserves [1]. Even with similar neurodegenerative changes, individuals vary considerably in their severity of cognitive aging [2]. Understanding potential interactions between social and biological processes, using a life-course perspective, is important to advancing potential causal explanations of disease onset and progression.

Vascular disease has been reported to be associated with cognitive impairment [3], as has having no leisure activities, childhood adversity, being in a lower socio-economic group, having less education, lower intelligence test scores, smoking, being female and older age [2, 4–13]. Relations between cognitive function and education [14, 15], as well as gender [16, 17] and alcohol use [18, 19], are not conclusive. For example, while education is associated with cognitive function, it is not always associated with rate of cognitive decline [15]. Longitudinal analyses have also indicated that those with different levels of education have similar brain pathology, but those with more years of education are better able to compensate for the effects of dementia [13].

Research across disciplines has indicated that physical activity is associated with lower risks of cognitive impairment [20–22]. Physical activity sustains cerebral blood flow by decreasing blood pressure, lowering lipid levels, inhibiting platelet aggregation or enhancing metabolic demands, and may improve aerobic capacity and cerebral nutrient supply [20]. However, engaging in physical activity is a marker of better health status, itself associated with lower risk of cognitive impairment and dementia.

Potential health protectors include social support (interactive processes whereby emotional, instrumental or financial aid is obtained from social network members) and the distinct concepts of civic engagement (ways in which people participate in their communities to improve lives or shape the community) and social capital (opportunities within communities to increase social resources through involvement in social, leisure, recreational activities, voluntary work, group membership, political activism, education) [23–25]. A small number of surveys have indicated that social integration, social engagement, and having strong networks are associated with better cognitive outcomes [26, 27] along with social and physical participation [6, 28]. For example, Fratiglioni et al. [26] combined four social network variables into an index, and reported that a poor or limited social network significantly increased the risk of dementia, with a significant gradient found for the four degrees of social connections. Read and Grundy [29] analysed data from the English Longitudinal Study of Ageing and reported poorer cognition in childless people, suggesting that there may be benefits to cognitive function from rearing and nurturing children. Singh-Manoux et al. [30], in cross-sectional analyses of phase 5 of the Whitehall II study, reported that, controlling for socio-economic status, participation in cognitively complex or socially oriented leisure activities had independent associations with cognitive status in middle age groups. Activities high on social engagement had a stronger and more consistent association with cognition than individual leisure activities. Singh-Manoux et al referred to other research indicating that active leisure is associated with adult cognition after adjusting for previously measured cognitive status [6].

Despite heterogeneity in study design and measures, a systematic review of the literature on social relationships and cognitive decline reported meta-analyses which showed that multiple aspects of social relationships are associated with cognitive decline [31]. In relation to such associations, the concept of a ‘mental bank’ has been coined, which can be increased or decreased by life experiences, and includes cognitive and affective resources (skills cognitive flexibility, effectiveness in learning, intelligence emotional or social skills and resistance to stress) [32]. These studies indicate the types of public health interventions that might improve cognitive health. Beddington et al. [33] argued, countries must learn how to capitalize on their citizens' cognitive resources if they are to prosper, both economically and socially, and suggested that early interventions will be key.

Theoretical frameworks for causal mechanisms include the effects of social and mentally stimulating interaction and participation, which may preserve cognitive function via activating thinking and attention [34]. This theory allows for people with higher cognitive reserve to avoid showing symptoms of cognitive decline for longer periods than those with lower cognitive reserve [13]. Social interaction requires many behaviours requiring cognitive skills (memory, attention, control) [35].

Social relationships may also provide stress buffering resources via the provision of informational, emotional, tangible and companionship support, by facilitating connectivity within the social network, and enhancing social integration [36]. Social relationships may also facilitate participation in social and other activities, thereby enhancing a self-concept of usefulness, of having a social role in life, self-esteem and identity, and maintainings a sense of self-efficacy, as well as provision of information (e.g. about health) [37, 38]. Participation in productive, civic or social activities may enhance one's self-concept of being useful, thereby increasing or maintaining self-esteem, identity, and self-efficacy. Szreter and Woolcock [39] pointed to the vast amount of research indicating that social capital is linked to enhanced well-being, reported mental and physical health, positive health behaviours, reduced levels of stress, loneliness and isolation. Such social resources have long been hypothesised to directly or indirectly promote a person’s adaptive behavioural responses to stress [40]. In relation to biomedical pathways, Lacey et al. [41] reported an association between social isolation and stress biomarkers (C-reactive protein). However, the literature also indicates that certain lifestyle factors which might be expected to increase cortisol secretion actually lead to a levelling of cortisol levels, suggesting that cortisol is less indicative of stress than expected, and that other stress biomarkers (including fibrinogen) may have a role [42, 43]. The need to examine associations between social resources and cognitive function further, and using a life-course approach, led to the study reported here.

## Aim

The aim of this study was to investigate the influence of life-course indicators of social engagement in civic activities on cognitive status at age 50, controlling for potential influences of early-life cognition (age 11), social networks and support, physical and mental health, health behaviours, socio-demographic and socio-economic characteristics.

## Methods

### Study data

The study used data from the British National Child Development Study (NCDS), a prospective cohort study originating in the Perinatal Mortality Survey [44]. The latter examined social and obstetric factors associated with still birth and infant mortality among over 17,400 babies born in Britain in 1 week in March 1958. Surviving members of this birth cohort were followed up on nine occasions in order to monitor changes in health, education, social and economic circumstances. The follow-ups were in 1965 (age 7), 1969 (age 11), 1974 (age 16), 1981 (age 23), 1991 (age 33), 1999/2000 (age 41/2), 2004–2005 (age 46/47), 2008–2009 (age 50), and a sequential mixed-methods follow-up in 2013 (age 55). Data about educational development, health behaviours, physical development, well-being, family life, economic circumstances, employment, social participation and attitudes towards life were collected. There have also been sub-sample surveys of the cohort. For example, participants were contacted at age 20 to map their examination achievements; and at age 44 to collect biomedical markers. Further information about the NCDS can be found on the Centre for Longitudinal Studies website (www.cls.ioe.ac.uk/ncds). Data for the NCDS sweeps are accessible (http://www.cls.ioe.ac.uk/ncds). The initial response rate to NCDS was just over 98% of all births in Great Britain in that week; although responses to subsequent waves varied (see Additional file 1). Power and Elliot [45] described respondent profiles.

### Sample

Sample members who completed NCDS surveys at both ages 11 and 50 were eligible for inclusion in the analyses reported here (*n* = 9119). Of these, 8129 (89.1%) completed the cognitive tests at both ages. Their survey data collected at ages 11, 33, 42 and 50 was analysed and is presented here. Cognitive results were imputed for 990 individuals (for age 11 or 50, or both); all 9119 were included in the analysis.

Age 11 was selected because the range of cognitive tests was wider. General Cognitive Ability was assessed at age 11 and not age 16, and most of those who were present in the study at 50 were present also at age 11, optimising the sample size for analysis (14,126 cohort members completed the age 11 tests, but far fewer, 11,920, completed the age 16 English and maths tests). Age 11 cognitive tests also feature prominently in the literature [46–52]. Ages 33, 42 and 50 were selected for analysis because these were the principal adult survey sweeps of NCDS (i.e. NCDS5, NCDS6 and NCDS8), and questions were included which measured the variables of interest here.

### Measures

Cognitive status at age 50 was the dependent variable, measured with memory and executive functioning tests, which have been widely used in surveys, and well tested [4, 53]. Memory was assessed by a word-recall test, involving memorising words with immediate and delayed recall. Respondents could score between 0 and 10 in both immediate and delayed recall tests, reflecting number of words remembered (thus higher scores reflected better performance). The overall score is calculated as sum of both recall tests, ranging between 0 and 20. Executive functioning was measured by letter cancellation and naming tests. Naming as many words from a particular category was used to test verbal fluency, and letter cancellation was used to test visual attention, speed and concentration. Respondents were asked to name as many animals as possible within one minute. In the letter cancellation test, respondents were asked to cross as many P’s and W’s as they could spot in the list of letters within one minute (maximum: 69); letter accuracy is the number of letters missed in the text during a test, with a lower score equating with a better result (the polarity was reversed to enable summing the standardised scores). Each test score was standardised to allow comparisons between all tests, and overall cognitive score was calculated by summing the standardised scores from each individual test.

Independent variables were selected according to their theoretical importance in the literature and comparable questions being repeated between waves. The influence of civic engagement and social activities on cognitive status at age 50 was examined by number and type of civic group activities currently participated in (ages 33, 50): membership of political party, trade union, environmental group, parents school association, residential group and neighbourhood watch, religious group or church organization, voluntary service group, other community, civic group, social/working men’s club, sports club, women’s institute/Townswomen’s Guild, women’s group, feminist organisation, professional organization, pensioners group/organization *(actual question wording),* scouts/guides organization, or others) formed a derived variable about civic engagement. In addition, other social activities measured at age 50 included visits to theatres, concerts, cinema, live sport events or pub/restaurant. A variable was created to represent the total number of civic activities engaged in by respondents at certain ages. This was derived at age 33 using reported numbers of civic activities engaged in (political party, charity/environmental groups, school/parental organizations, neighbourhood/residents associations, and women’s institutes/groups); and age 50 respondents were asked separately for each type of civic activity and positive answers were then summed to provide the total number of civic activities engaged in.

The independent variables analysed as potential confounders included early-life cognition (age 11), social networks and support, physical and mental health, health behaviours, socio-demographic and socio-economic characteristics:

Cognitive ability at age 11: Cognitive tests at age 11 were used to measure child cognitive ability: reading, mathematics, copying designs and general ability. The Reading Comprehension Test had scores between 0 and 35, Arithmetic/Mathematics Test between 0 and 40, Copying Design Test (in which children copied 6 objects, each twice) between 0 and 12 and General Ability Test (consisting of 40 verbal and non-verbal tasks, tested by their teachers, designed by the National Foundation for Educational Research [54] between 0 and 80. As with cognition at age 50, each score was standardised to allow comparison between tests, and overall cognitive score at age 11 was derived by summing the standardised scores of all four tests. For cognition at age 11 and at age 50, categorical variables were also constructed by dividing standardised continuous scores using cut-offs of –0.5 S.D. and +0.5 S.D. and creating ‘below mean’, ‘mean’ and ‘above mean’ categories of cognitive status at both ages [4]. An additional variable representing cognitive change was constructed as a change between cognition categories at ages 11 and 50. The cognitive tests included at age 11, are widely used and have been validated in several longitudinal studies: reading comprehension: [55], maths test [56], copying designs test: [57], general ability test [54].

Social networks and support: questions on sources of advice about important changes in life (age 33); whether they had someone to turn to for advice/support, and, if so, who (ages 42, 50); a social network variable was derived from the latter two questions (having someone to turn to for advice/support, and who), equating to whether anyone was available for advice/support, and who that person was; having someone who would listen to their problems; whether they visited/were visited/had phone/mail contacts with friends in last 2 weeks (age 50); marital/partnership status (ages 33, 42, 50), household size (ages 33, 50), and had help or advice from friends/neighbours/colleagues and family members (ages 33, 42, 50). Those in relationships were asked whether they assessed their relationship as a happy one, and ratings of ‘how happy’ (ages 33, 42, 50) (question type/wording varied slightly by wave).

Health behaviour: questions on participation in sporting activities, and its frequency at ages 33, 42 and 50; alcohol consumption and frequency at ages 33, 42 and 50; current smoking status, and frequency, at ages 42 and 50. Obesity was measured by body mass index at ages 33 and 42. Physical health: Self-reported health status at ages 33 and 50; reported fits/epilepsy at ages 33, 42 and 50; biomarkers and measurements at age 44, including serum cholesterol, triglycerides, low density lipoprotein, high density lipoprotein, blood pressure and waist circumference. Mental health: psychiatric morbidity was measured with the Malaise Index (the 9-item Malaise Inventory was analysed) [58] at ages 33, 42 and 50. This was developed from the Cornell Medical Index (also referred to as mental well-being). Each positive response to the nine items is scored as one, with a total score ranging between 0 and 9, with higher scores indicating worse mental health. Additionally, the score was dichotomised with scores of 4+ indicating poorer health.

Standard socio-demographic characteristics included gender, marital/partnership status, highest level of qualification by age 50, housing tenure in childhood (ages 7 and 11); socio-economic position: life-course social class, using the six standard Registrar General’s categories (father’s social class, as reported by parents, at respondents’ birth, and at ages 7 and 11; respondent’s self-reported social class (at ages 42 and 50). At age 50, current employment was included as an indicator of socio-economic activity. The question wording of variables included in the final model is given in Additional file 2.

## Analyses

The distributions of variables were examined with univariate statistics; bivariate analyses were conducted to test associations between independent and the dependent variables. Variables which were significantly associated with the dependent variable at least at the 0.05 level of statistical confidence, or which were of border-line significance, in bivariate analyses were included in fully adjusted, multi-variable analysis (see variables in Additional file 3).

Multiple linear regression analysis was used to examine the independent influence of the independent variables on cognitive status at age 50. Hierarchical regression was selected as the method of variable entry as it is theory- rather than data-driven. No inter-correlation was higher than r = 0.40, indicating that multicollinearity was at an acceptable level, permitting variable entry.

Complete case and multiple imputation analysis were conducted. Missing information was imputed by Multiple Imputation by Chained Equations for the final model [59] to deal with reduced sample size over the NCDS waves and boost analytical power. The uncertainty from estimating imputed values is accounted for in standard error estimates. The method used was multiple imputation. The method was used for those who were present in the study at ages 11 and 50. In this imputation all variables identified in the final model of complete case analysis and additional variables predictive of missingness were included. Ten imputed datasets were created. Data were analysed using STATA 13.0. It should be noted that missing cases for the biomedical variables appeared to be ‘not-at-random’ (possibly because of higher non-responses to items and nurse follow-up interviews to collect these data) so biomedical information could not be used in the imputed analyses and were thus withdrawn from the analyses.

## Results

### Comparisons between original sample and analytical sample presented here

The analytical sample included 49% males and 51% females, compared to the ‘birth’ sample of 52% males and 48% of females. There were no differences between their distributions of highest achieved qualification nor marital/partnership status (age 50). Slightly more rated their health status as excellent in the analytical sample, compared with the cross-sectional sample at age 33, but the difference was small (1%); there were no differences in self-reported health status at age 50. The mean (S.D.) for the reading scores at age 11 was 16.7 (6.1) in the analytical sample and 16.0 (6.3) in the cross-sectional sample; for the maths scores these were 17.9 (10.2) and 16.6 (10.4); for the copying scores 8.4 (1.4) and 8.3 (1.5); and for the General Ability Test 45.3 (15.5) and 42.9 (16.1) (all respectively). The mean (and S.D.) results for the cognitive test scores at age 50 were almost identical in both samples: letter accuracy: 4.40 (4.11) in the analytical sample and 4.42 (4.12) in the cross-sectional sample; animal naming: 22.32 (6.30) and 22.28 (6.30); word recall immediate: 6.54 (1.49) and 6.54 (1.49); word recall delayed: 5.41 (1.84) and 5.41 (1.84) (all respectively).

### Characteristics of the analytical sample

The sample characteristics are shown in Table 1 (and see Additional file 3 referenced earlier). At age 11, 6% of child respondents’ fathers were in professional social classes, 19% managerial-technical, 10% skilled non-manual and the remainder manual. At age 42, 6% of participants were classified as professional, 39% as managerial-technical, 22% skilled non-

**Table 1:** Description of the sample and variables used in the analysis

		Number	Frequencies (%)/Mean (S.D)	% missing
Cognition				
Standardized score at age 11		8448	0.44 (3.12)	7.4
Standardized score at age 50		8751	0.02 (2.41)	4.0
Social network				
Age 33				
Has at least 1 friend/ neighbour/ colleague could turn to for advice (Number of people showed only for description; variable used as dichotomous)		7961		12.7
	No/No one mentioned		56.3	
	Yes/Someone mentioned		43.7	
	0 people mentioned		56.3	
	1 person mentioned		28.2	
	2 people		12.2	
	3 people		2.7	
	4 people		0.3	
Has at least 1 member of family could turn to for advice (Number of people showed only for description; variable used as dichotomous)		7961		12.7
	No/No one mentioned		8.7	
	Yes/Someone mentioned		91.3	
	0 people mentioned		8.7	
	1 person mentioned		21.8	
	2 people		25.5	
	3 people		28.2	
	4 people		15.8	
Number of civic group activities participated in (used as continual variable; categories showed only for description)		7961	0.22 (0.52)	12.7
	No activity		82.7	
	1 activity		13.8	
	2 activities		2.8	
	3+		0.7	
Age 42				
Has somebody could turn to for advice/support		8641		7.2
	No		3.3	
	Yes, family member		75.0	
	Yes, friend/neighbour/colleague		21.7	
Age 50				
Number of civic group activities participated in (used as continual variable; categories showed only for description)		9117	0.49 (0.80)	0.1
	No activity		65.1	
	1 activity		24.6	
	2 activities		7.5	
	3+		2.8	
Health and health behaviour				
Age 33				
Self-rated health		7843		14.0
	Poor		1.3	
	Fair		11.1	
	Good		52.2	
	Excellent		35.4	
Age 42				
Mental well-being (Malaise score; 9-item version)		8412		7.8
	Better (0–3)		88.5	
	Worse (4+)		11.5	
Frequency of drinking alcohol		8459		
	Never		1.2	
	Not now/ special occasions		16.0	
	Once in a week		29.9	
	More often		52.9	
Frequency of smoking		8464		7.2
	No		46.1	
	Used to/ occasionally		30.2	
	Daily		23.7	
Takes part in sporting activities and frequency		8455		7.3
	Not regularly/less often than once in month		27.7	
	2–3 times in month		6.5	
	Once in week		18.8	
	2–3 times in week		21.2	
	4 times in week/every day		25.8	
Socio-economic background				
Age 11				
Father’s (male head) social class		8249		9.5
	Professional		5.8	
	Managerial-technical		19.2	
	Skilled non-manual		9.5	
	Skilled manual		40.2	
	Partly skilled		15.8	
	Unskilled		5.0	
	No male head		4.5	
Housing tenure		8342		8.6
	Owner occupied		48.1	
	Council rented		39.8	
	Private rented		7.3	
	Rent free		4.8	
Age 42				
Own social class		7297		20.0
	Professional		5.6	
	Managerial-technical		38.9	
	Skilled non-manual		21.6	
	Skilled manual		19.2	
	Partly skilled		11.9	
	Unskilled		2.8	
Age 50				
Highest achieved qualification		9113		0.1
	None		11.0	
	CSE or equivalent		11.2	
	GCSE or equivalent		25.4	
	AS/A level or equivalent		17.2	
	Degree/teaching diploma/vocational NVQ4 diploma		30.8	
	Higher Degree/vocational NVQ5 diploma		4.4	
Other				
Gender		9119		0
	Male		49.0	
	Female		51.0	

manual and the remainder manual. At age 50, 4% of respondents reported having a Higher Degree/vocational NVQ5 Diploma (National Vocational Qualifications range from Level 1 focusing on basic work activities to Level 5 for senior management), 31% had achieved a Degree/Teaching Diploma/vocational NVQ4 Diploma, 17% had Advanced General Certificate of Secondary Education (AS/A-levels) or equivalent qualifications, 25% had General Certificate of Secondary Education (GCSE) or equivalent qualifications, 11% had Certificate of Secondary Education (CSE) or equivalent qualifications; and 11% had no qualifications.

The continuous distributions of all cognitive tests at ages 11 and 50 were approximately normal. Categorically, at age 11, 28% of respondents were classified in the ‘below the mean’ category, 35% in the ‘mean’ category, and 37% in the ‘above the mean’ category. At age 50, the comparable percentages were 31, 39 and 30% respectively. Cognitive score changes between ages 11 and 50 show that almost a third of the analytical sample’s cognitive scores deteriorated between ages 11 and 50 (with over 6% showing deterioration over two levels (meaning scoring ‘above the mean’ at age 11 and scoring ‘below the mean’ at age 50) and 25% deteriorated by one level (either from ‘above the mean’ at age 11 to ‘at the mean’ at age 50, or from ‘at the mean’ at age 11 to ‘below the mean’ at age 50). Under half of participants, 44%, had unchanged scores (in the same category) at both ages and a quarter achieved better results at age 50 (almost 20% improving by one category and almost 5% improving by 2 categories) (Additional file 4).

Most (83%) of respondents at age 33, and 64% at age 50, reported no participation in any civic organisation. Participating in one civic organisation was reported by 14% of respondents at age 33 and by 25% at age 50.

Table 2 shows the crude bivariate associations between standardized cognitive scores at age 50 and potential predictive variables, as estimated by linear regression (at least at 0.05 level, or achieving borderline significance). Those with higher level achieved qualifications at age 50 had the strongest positive association with cognition at age 50 (those respondents who reported having AS/A-levels/diploma/degree achieved 1.4 to 2.6 points higher cognitive scores, compared to those with no qualification); those with good or excellent self-rated health at age 33 had 0.7 to 1.0 higher cognitive scores; those who participated in civic group activities at ages 33 and 50 scored 0.4 to 0.6 more in cognitive tests; and those who took part in sporting activities achieved between 0.4 to 0.6 higher cognitive scores. An inverse association was found with father’s social class and own reported social class (those whose fathers were in manual groups scored 1.2 to 1.7 lower, compared with those whose fathers were in professional classes; those who reported themselves in manual classes at age 42 scored 1.7 to 2.1 lower).

**Table 2:** Bivariate associations between standardized cognitive score at age 50 and predictive variables over the life-course (linear regression)

		Unstandardized B Standardized β	95% CI (*p*-value)	t-test
Cognition in childhood				
Standardized score at age 11	Per unit	0.29 0.36	0.27 to 0.30 (<0.0001)	35.0
Social network				
Age 33				
Has at least 1 friend/ neighbour/colleague could turn to for advice	No / No one mentioned		0 (ref)	
	Yes	0.41 0.08	0.30 to 0.51 (<0.0001)	7.70
Has at least 1 member of family could turn to for advice	No/ No one mentioned		0 (ref)	
	Yes	0.46 0.08	0.34 to 0.58 (<0.0001)	7.50
Number of civic group activities participated in	Per 1 activity +	0.56 0.12	0.45 to 0.65 (<0.0001)	11.00
Age 42				
Has somebody could turn to for advice	No		0 (ref)	
	Family member	0.60 0.11	0.31 to 0.90 (<0.0001)	4.01
	Friend/colleague/neighbour	0.64 0.11	0.33 to 0.95 (<0.0001)	4.03
Age 50				
Number of civic group activities participated in	Per 1 activity +	0.39 0.13	0.32 to 0.45 (<0.0001)	12.13
Health and Health behaviour				
Age 33				
Self-rated health	Poor		0 (ref)	
	Fair	0.23 0.03	–0.28 to 0.75 (0.38)	0.88
	Good	0.73 0.15	0.24 to 1.22 (0.004)	2.91
	Excellent	0.98 0.20	0.41 to 1.48 (<0.0001)	3.87
Age 42				
Mental well-being (Malaise score; 9-item version)	Better (0–3)		0 (ref)	
	Worse (4+)	–0.45 –0.06	–0.61 to –0.29 (<0.0001)	–5.41
Frequency of drinking alcohol	Never		0 (ref)	
	Not now/ special occasions	–0.09 –0.1	–0.60 to 0.41 (0.72)	–0.35
	Once in a week	0.11 0.02	–0.38 to 0.61 (0.66)	0.44
	More often	0.56 0.11	0.07 to 1.06 (0.03)	2.23
Frequency of smoking	No		0 (ref)	
	Used to / occasionally	–0.08 –0.01	–0.20 to 0.04 (0.21)	–1.24
	Daily	–0.58 –0.10	–0.71 to –0.44 (<0.0001)	–8.63
Takes part in sporting activities and how frequently	Not regularly/ less often than once in month		0 (ref)	
	2–3 times in month	0.51 0.05	0.28 to 0.74 (<0.0001)	4.46
	Once in week	0.52 0.08	0.36 to 0.67 (<0.0001)	6.55
	2–3 times in a week	0.58 0.10	0.43 to 0.73 (<0.0001)	7.61
	4 times in week/every day	0.40 0.07	0.25 to 0.54 (<0.0001)	5.43
Socio-economic background				
Age 11				
Father’s social class	Professional		0 (ref)	
	Managerial-technical	–0.40 –0.07	–0.64 to –0.15 (0.002)	–3.17
	Skilled non-man	–0.65 –0.08	–0.93 to –0.38 (<0.0001)	–4.68
	Skilled manual	–1.15 –0.23	–1.38 to –0.92 (<0.0001)	–9.75
	Partly skilled	–1.35 –0.20	–1.60 to –1.09 (<0.0001)	–10.46
	Unskilled	–1.66 –0.15	–1.98 to –1.34 (<0.0001)	–10.25
	No male head	–1.04 –0.09	–1.37 to –0.71 (<0.0001)	–6.23
Housing tenure	Owner occupied		0 (ref)	
	Council rented	–0.75 –0.15	–0.86 to –0.64 (<0.0001)	–13.12
	Private rented	–0.48 –0.05	–0.69 to –0.28 (<0.0001)	–4.60
	Rent free	–0.31 –0.03	–0.56 to –0.06 (0.02)	–2.42
Age 42				
Own social class	Professional		0 (ref)	
	Managerial-technical	–0.51 –0.10	–0.75 to –0.26 (<0.0001)	–4.09
	Skilled non-man	–0.96 –0.17	–1.21 to –0.70 (<0.0001)	–7.34
	Skilled manual	–1.71 –0.28	–1.97 to –1.45 (<0.0001)	–12.97
	Partly skilled	–1.55 –0.21	–1.83 to –1.28 (<0.0001)	–11.04
	Unskilled	–2.11 –0.15	–2.50 to –1.71 (<0.0001)	–10.43
Age 50				
Highest achieved qualification	None		0 (ref)	
	CSE or equivalent	0.41 0.05	0.20 to 0.62 (<0.0001)	3.89
	GCSE or equivalent	0.96 0.17	0.78 to 1.14 (<0.0001)	10.56
	AS/A level or equivalent	1.37 0.22	1.18 to 1.56 (<0.0001)	14.24
	Degree/teaching diploma/vocational NVQ4 diploma	2.07 0.40	1.89 to 2.24 (<0.0001)	23.46
	Higher Degree/vocational NVQ5 diploma	2.63 0.22	2.35 to 2.91 (<0.0001)	18.62
Gender	Male		0 (ref)	
	Female	0.47 0.10	0.37 to 0.57 (<0.0001)	9.08

Further bivariate regression analyses showed that each individual type of civic activity at age 33 had a significant and positive effect on cognitive status at age 50 (active member of political party: B = 0.97, 95% CI 0.61 to 1.34, *p*-value < 0.001; active in charity activities: 0.97; 0.81 to 1.15; <0.001; active in women’s organizations: 0.81; 0.46 to 1.16; <0.001; active in neighbourhood watch: 0.63; 0.29 to 0.96; <0.001; active in school/parental organisaton: 0.64; 0.40 to 0.88; 0.001). Although there were some small differences between individual regression coefficients, confidence intervals substantially overlapped, and differences between effects of different activities were not statistically significant.

### Multivariable analyses

Using the imputed dataset, multiple linear regression analysis was conducted to assess the independent influence of those variables identified in bivariate analysis as potential predictors. Table 3 shows the results of the fully-adjusted model. Participation in civic organizations, clubs or groups at ages 33 and 50 both retained significant associations with cognition at age of 50 (participation in each additional civic activity increased cognitive scores by, on average, 0.12 points).

**Table 3:** Multiple linear regression; association between predictors and cognitive status at age 50 (data imputed for missing cases; estimated model)

		Stand-ard-ised β	95% CI (*p*-value)	t-test
Cognition in childhood				
Standardized score at age 11	Per unit	0.19	0.17 to 0.21 (<0.0001)	18.73
Social network				
Age 33				
Has > = 1 friend/neighbour/ colleague could turn to for advice (ref = 0 ‘No/No one mentioned)	Yes	0.10	–0.04 to 0.17 (0.16)	1.42
Has at least 1 member of family could turn to for advice (ref = 0 ‘No/No one mentioned)	Yes	–0.11	–0.18 to 0.08 (0.02)	–2.32
Number of civic group activities participated in	Per 1 activity +	0.12	0.02 to 0.22 (0.03)	2.22
Age 42				
Has somebody could turn to for advice (ref = 0 ‘Nobody’)	Family member	–0.01	–0.30 to 0.27 (0.93)	–0.09
	Friend/colleague/neighbour	0.02	–0.27 to 0.32 (0.89)	0.15
Age 50				
Number of civic group activities participated in	Per 1 activity more	0.13	0.07 to 0.20 (<0.0001)	4.34
Health and Health behaviour				
Age 33				
Self-rated health (ref = 0 ‘Poor’)	Fair	0.01	–0.49 to 0.50 (0.99)	0.01
	Good	0.14	–0.36 to 0.64 (0.58)	0.56
	Excellent	0.16	–0.35 to 0.68 (0.52)	0.64
Age 42				
Mental well-being (Malaise score; 9-item version) ref = 0 ‘Better 0/3’	Worse (4+)	–0.18	–0.33 to –0.02 (0.03)	–2.23
Frequency of drinking alcohol (ref = 0 ‘Never’)	Not now/Special occasions	0.11	–0.39 to 0.60 (0.67)	0.42
	Once a week	0.18	–0.32 to 0.67 (0.49)	0.71
	More often	0.30	–0.21 to 0.81 (0.24)	1.18
Frequency of smoking (ref = 0 ‘No)	Used to/ occasionally	0.07	–0.04 to 0.19 (0.21)	1.22
	Daily	0.03	–0.1 to 0.17 (0.61)	0.51
Takes part in sporting activities and how frequently (ref = 0 ‘Not regularly’)	2–3 times a month	0.16	–0.05 to 0.38 (0.13)	1.50
	Once a week	0.15	0.01 to 0.30 (0.03)	2.14
	2–3 times a week	0.19	0.05 to 0.33 (0.01)	2.59
	4 times week / everyday	0.18	0.05 to 0.31 (0.006)	2.73
Socio-economic background				
Age 11				
Father’s (male head) social class (ref = 0 ‘Professional’)	Managerial-technical	–0.17	–0.42 to 0.07 (0.16)	–1.41
	Skilled non-manual	–0.24	–0.53 to 0.05 (0.09)	–1.70
	Skilled manual	–0.22	–0.47 to 0.01 (0.05)	–1.94
	Partly skilled	–0.27	–0.52 to –0.03 (0.03)	–2.21
	Unskilled	–0.29	–0.58 to 0.02 (0.06)	–1.90
	No male head	–0.10	–0.41 to 0.24 (0.57)	–0.57
\Housing tenure (ref = 0 ‘Owner occupied’)	Council rented	–0.03	–0.15 to 0.08 (0.51)	–0.66
	Private rented	0.02	–0.17 to 0.22 (0.82)	0.23
	Rent free	0.06	–0.21 to 0.25 (0.63)	0.48
Age 42				
Own social class ((ref = 0 ‘Professional’)	Managerial-technical	–0.06	–0.28 to 0.16 (0.61)	–0.50
	Skilled non-manual	–0.17	–0.43 to 0.09 (0.19)	–1.27
	Skilled manual	–0.28	–0.54 to –0.03 (0.03)	–2.17
	Partly skilled	–0.32	–0.60 to –0.04 (0.02)	–2.22
	Unskilled	–0.38	–0.76 to –0.004 (0.05)	–1.96
Age 50				
Highest achieved qualification (ref = 0 ‘None’)	CSE or equivalent	0.23	0.003 to 0.41 (0.03)	2.16
	GCSE or equivalent	0.33	0.15 to 0.51 (<0.0001)	3.61
	AS/ A-level or equivalent	0.61	0.42 to 0.80 (<0.0001)	6.27
	Degree/teaching diploma/vocational NVQ4 diploma	0.83	0.64 to 1.02 (<0.0001)	8.44
	Higher degree/NVQ5 diploma	1.08	0.79 to 1.38 (<0.0001)	7.17
Gender (ref = 0 Male)	Female	0.49	0.38 to 0.60 (<0.0001)	9.22
Constant		–0.90	–1.66 to –0.14 (0.02)	–2.32
R2		0.1767		
Adjusted R2		0.1730		
F statistic (40, 6463.0)		42.47		
* P*-value		<0.0001		

Support from family at age 33 was inversely associated with cognition at age 50: having at least one family member to whom respondent could turn to for advice at age 33 was associated with a decreased cognitive score at age 50 by 0.11 points. Support from friends at ages 33 and 42, respectively, did not retain statistical significance, as their influence was explained by the other variables included in the regression model.

Those who at age 33 reported their health as good-excellent had slightly higher cognitive scores by 0.14–0.16 points at age 50, compared with those whose self-reported health was poor (reference category) This was not statistically significant and, as Table 3 shows, the 95% confidence interval was wide, ranging from -0.36 to 0.64. Those who registered 4 or more on the Malaise Index (indicating worse mental well-being) at age 42 had on average 0.18 lower cognitive score at age 50 than those who scored 0–3.

The association of participation in sport (and frequency) at age 42 with later cognitive outcomes showed a positive effect for those who participated in sport at least weekly. The latter had 0.15–0.19 higher overall cognitive scores compared with those who participated in sport less often or not at all. Associations between frequency of drinking alcohol, smoking cigarettes and cognitive scores at age 50 were fully explained by other variables in the final model.

The effect of socio-economic characteristics in childhood (father’s socio-economic position and housing tenure at age 11) was fully explained in the final model. Own social class at age 42 was negatively significantly associated with cognition at age 50, and those in manual social classes (skilled, partly skilled, unskilled) had 0.29–0.38 points lower overall cognitive scores compared with those in non-manual classes. Higher cognitive scores at age 50 were achieved by those with higher-level qualifications (showing stepwise increase by 0.23 to 1.08 points compare to those who did not achieved any qualification). Females had, on average, 0.49 points higher cognitive scores than males.

In summary, the model shows that cognitive status at age 11, participation in civic activities (ages 33 and 50), frequent participation in sport (age 42), having higher level qualifications by age 50, and female gender were positively and significantly associated with cognitive outcomes at age of 50. Having a father in manual socio-economic groups at age of 11, reporting oneself to be in a manual group, and higher Malaise Index scores (age 42) were negatively associated with cognitive outcomes at age of 50. Multiple regression analysis, with age 50 cognitive status as the dependent variable, showed that the overall model was highly significant, and explained approximately 18% of the variance in cognitive scores at age 50.

## Discussion

This study investigated associations between life-course social engagement (civic participation) and cognitive status at age 50, adjusting for potential confounders. Our approach aimed to be original by using a large, British longitudinal birth cohort (NCDS), which enabled us to take into account complex interactions between social and biological processes, thus employing a life course perspective at multiple time-points. It was pointed out in the Background that a small number of surveys have indicated that social integration, engagement and participation, and having strong networks are associated with better cognitive outcomes, although relatively few studies have used life course data.

Age 11 was selected for analysis because the range of cognitive tests was wider, and inclusion of age 11 rather than age 16 optimised the sample size for analysis. Ages 33, 42 and 50 were selected for analysis because these were the principal adult survey sweeps of NCDS, and questions were included which measured the variables of interest. Fully-adjusted analyses showed that those variables which were positively and significantly associated with cognitive status at age 50 were: cognitive ability at age 11, participation in civic activities (including clubs, groups) (ages 33, 50), frequent engagement in sport (age 42), better (i.e. lower) Malaise Index scores (age 42), having higher level qualifications, and female gender. Socio-economic indicators at ages 11 and 42 were inversely associated with cognitive status at age 50. The proportion of explained variance in the regression model (18%), while modest, is relatively impressive given the multi-faceted causality of cognitive ability. Thus the results reported here indicated modest longitudinal associations between adult social engagement and cognitive function at age 50, which persisted after adjusting for covariates. The implication is that if people continue to engage throughout life, maintaining related social skills, there may be some protection from cognitive decline.

Despite the literature indicating the importance of having strong social networks and support for optimum mental and physical health outcomes, and for reducing mortality risk [24, 26, 60], it is uncertain why support from family, but not from friends, was inversely associated with cognitive scores in this study. It would be expected from this literature that support from family at least would be positively associated with physical and mental health outcomes, especially as relatives are more likely than friends to provide instrumental and informational support [61, 62].

The strength of the study was its longitudinal nature, based on a large, national British cohort of males and females: the National Child Development Study. A limitation of the study was that only memory and executive function were tested, their measurement was partial, and the measures were non-conventional neuropsychological tests. As with any longitudinal study, and despite excellent initial response rates, differential patterns of response in NCDS over the life course may lead to a danger of attrition bias in a complete-case analysis [63, 64]. In the 39 years between the cognitive tests at age 11 and age 50 we would, for example, expect to lose a slightly disproportionate number of men, those from lower SES backgrounds, those with less good health and those with lower cognitive skills/qualifications. To correct for this, a process of multiple imputation by chained equations (MICE) was employed. The plausibility of the Missing at Random assumption was maximised [65], and the imputation process was in line with its assumptions [66]. Thus the imputed population at age 50 had the same basic characteristics as those at age 11 (e.g. the analytical sample did not ‘lose’ more people with lower (age 11) cognitive powers in the intervening years than those with higher cognitive powers).

## Conclusions

In conclusion, this paper contributes to the body of literature on potential behavioural risk factors for cognitive decline [22, 67], and on the benefits of civic participation. Adult social engagement through civic activities could potentially maintain cognitive function at age 50, independently of behavioural and socio-economic circumstances, supporting theories of neuroplasticity. The direction of causality can, of course, be questioned. In the study reported here, cognitive status was assessed at age 11 (educational assessments), then not again until age 50 (cognitive function survey measures). The analyses controlled for potential confounding variables, including physical, biomedical, and mental health variables; only the Malaise Index was a significant predictor. It is possible that the sample was too young at age 50 for the full assessment of their long-term impact on cognitive status, or that the physical health variables were insufficiently sensitive. The findings require verification in future longitudinal surveys, using robust measures, and with the pertinent measures repeated at key waves.

While the limitations of this study preclude definitive conclusions, there is a case for causal interpretation of this association. There is a rich literature on how social factors might improve physical and psychological health and well-being, both directly and as stress buffers, for example via social comparisons of oneself with others’, perceptions of self-esteem and sense of control over life and identity [68]. Potential causal mechanisms in relation to cognitive function include the stimulation derived from social interaction and participation, with maintenance of social and communication skills, which might preserve cognitive function. In conclusion, potential modifiable targets for public health policy intervention in promoting cognitive health include encouragement of civic engagement and provision of opportunities for this, and modification of behavioural risk factors (encouragement of physical activity).

## Additional files

Additional file 1: Table S1. Response by wave. National Child Development Study (NCDS) longitudinal response by follow-up sweep number. Response rates to NCDS survey sweeps. (DOCX 19 kb)
Additional file 2: Table S2. Variable description. Question wording of variables included in the final model. (DOCX 23 kb)
Additional file 3: Table S3. Sample description bivariates. Description and bivariate association from linear regression between each theory-driven independent variable considered for entry and cognition at age 50. Description and bivariate association from linear regression analyses.
Additional file 4: Table S4. Change tables cognition. Distribution of categorised cognitive scores. Cognitive score changes between ages 11 and 50. (DOCX 18 kb)

## Abbreviations

ESRC: Economic and Social Research Council
NCDS: National Child Development Study
NVQ: National Vocational Qualifications (from Level 1 on basic work activities, to Level 5 senior management)
