# Supplementary material for: Is mid-life social participation associated with cognitive function at age 50? Results from the British National Child Development Study (NCDS)
Source: BMC Psychol. 2016 Dec 2;4:58. doi: 10.1186/s40359-016-0164-x (PMC5134123; doi:10.1186/s40359-016-0164-x)
Supplement: Additional file 1: Table S1. — Response by wave. National Child Development Study (NCDS) longitudinal response by follow-up sweep number. Response rates to NCDS survey sweeps. (DOCX 19 kb) [file 40359_2016_164_MOESM1_ESM.docx]

**Additional file 1 Table S1:**

**National Child Development Study (NCDS) longitudinal response by follow-up sweep number**

|  | PMS | NCDS1 | NCDS2 | NCDS3 | NCDS4 | NCDS5 | NCDS6 | Biomedical | NCDS7 | NCDS8 | NCDS9 |
| --- | --- | --- | --- | --- | --- | --- | --- | --- | --- | --- | --- |
|  | 1958 | 1965 | 1969 | 1974 | 1981 | 1991 | 2000 | 2002 | 2004 | 2008 | 2013 |
|  | Birth | 7 | 11 | 16 | 23 | 33 | 42 | 44 | 46 | 50 | 55 |
| Productive | 17,415 | 15,425 | 15,337 | 14,654 | 12,537 | 11,469 | 11,419 | 9,377 | 9,534 | 9,790 | 9,137 |
| Refusal |  | 80 | 797 | 1,151 | 915 | 1,365 | 1,148 | 2,830 | 1,448 | 1,214 | 582 |
| Non-Contact | 218 | 1,036 | 406 | 786 | 1,675 | 1,394 | 1,832 | 792 | 612 | 835 | 860 |
| Other unproductive |  | 173 | 202 | 295 | 413 | 953 | 263 | 31 | 109 | 332 | 491 |
| Ineligible |  | 0 | 0 | 0 | 0 | 0 | 13 | 65 | 11 | 81 | 0 |
| Not Issued | 925 | 548 | 275 | 0 | 862 | 993 | 1,416 | 2,908 | 4,249 | 3,554 | 4,543 |
| Not issued - emigrant |  | 475 | 701 | 799 | 1,196 | 1,335 | 1,268 | 1,234 | 1,272 | 1,293 | 1,286 |
| Not issued – dead |  | 821 | 840 | 873 | 960 | 1,049 | 1,199 | 1,321 | 1,323 | 1,459 | 1,659 |
| Total | 18,558 | 18,558 | 18,558 | 18,558 | 18,558 | 18,558 | 18,558 | 18,558 | 18,558 | 18,558 | 18,558 |

**Source: University of London. UCL Institute of Education. Centre for Longitudinal Studies. (2015). *National Child Development Study Response and Outcomes Dataset, 1958-2013*. [data collection]. *5th Edition.*UK Data Service. SN: 5560,**[**http://dx.doi.org/10.5255/UKDA-SN-5560-3**](http://dx.doi.org/10.5255/UKDA-SN-5560-3)**.**

**Key:**

**NCDS: National Child Development Study**

**PMS: Perinatal mortality survey**

**Note: As with any longitudinal study, differential patterns of attrition may lead to response bias in a completed-cases NCDS analysis.   Men are slightly less likely to remain in the study than women, as are those from lower SES backgrounds and those with lower basic skills.    But since the birth survey interviewed 98.5% of those born in GB in the specified week, the birth dataset profile provides an excellent basis for subsequent response-bias correction using inverse-probability weighting, Full Information Maximum Likelihood estimation or (as in the case of this paper) multiple imputation**.
