# Supplementary material for: Is mid-life social participation associated with cognitive function at age 50? Results from the British National Child Development Study (NCDS)
Source: BMC Psychol. 2016 Dec 2;4:58. doi: 10.1186/s40359-016-0164-x (PMC5134123; doi:10.1186/s40359-016-0164-x)
Supplement: Additional file 2: Table S2. — Variable description. Question wording of variables included in the final model. (DOCX 23 kb) [file 40359_2016_164_MOESM2_ESM.docx]

**Additional file 2: Table S2 Variable description**

Age 11 Father’s/male head socioeconomic position

Categories were labelled to match similar variable later in the life showing participant’s own SEP (at age 42):

Professional; managerial-technical; skilled non-manual; skilled manual; partly skilled; unskilled; and special category for ‘no male head’ at age 11

Age 11 Household tenure

Owner occupied, council rented, private rented, rent free

Age 33 & 50 Civic participation:

“Whether cohort member currently member of:

-political party

-trade union

-environmental group

-parents school association

-residential group and neighbourhood watch

-religious group or church organization

-voluntary service group

-other community, civic group

-social/ working men’s club

-sport club

-women’s institute/ Townswomen’s Guilds

-women’s group, feminist organisation

-professional organization

-pensioners group/ organization ^+^

-Scouts/ Guides organization

-others”

(^+^ exact phrase used in the questionnaire)

Age 33 Help

“Source of advice about important life change:

spouse/partner, parent/in-law, other relative, friend or neighbour, work colleague, church, charity, etc; someone paid to help, other source of help”

– possibility to give four answers

Information was summed up which gives number of family members and colleagues/friends/neighbours who are close to give advice ; variable then re-categorized whether participant has at least 1 family members and at least 1 friends /neighbours/colleagues

Age 42 Support:

“Does participant have someone (s)he could turn to for advice/support”

Y/N

“Who is the person cohort member could turn to for advice/support?”

One variable vas derived with 3 categories:

No

Family member: Spouse/partner; Mother; Father; Brother; Sister

Friend/colleague/neighbour: Boyfriend; Girlfriend; Female friend; Male friend; Neighbour;

Other

Age 42 Socioeconomic position

Higher managerial and professional occupations

Lower managerial and professional occupations

Intermediate occupation

Small Employers and Own account workers

Lower supervisory and technical occupations

Semi-routine occupations

Routine occupations

According to NS-SEC guide we have compound these into: professional, managerial-technical, skilled non-manual, skilled manual, partly skilled, unskilled ([*http://www.ons.gov.uk/ons/guide-method/classifications/current-standard-classifications/soc2010/soc2010-volume-3-ns-sec--rebased-on-soc2010--user-manual/index.html*](http://www.ons.gov.uk/ons/guide-method/classifications/current-standard-classifications/soc2010/soc2010-volume-3-ns-sec--rebased-on-soc2010--user-manual/index.html)*; accessed April 2015)*)

Age 42 Sport frequency variable:

Derived from 2 questions:

“Does participant do any regular exercise?” with categories Y/N

“How often participant takes part in any exercise activity?” with categories:

Every day

4-5 days a week

2-3 days a week

Once a week

2-3 times a moth

Less often

Derived into:

not regularly/less often than 1 in mth; 2-3 in mth, 1 in wk, 2-3 in wk, 4 in wk/ every day

Age 42 Frequency of drinking

(Q : ‘Frequency cohort member has alcoholic drink of any kind’ with 7 possible answers)

Then it was grouped into 4 categories:

Daily/nearly daily: On most days/2-3 days a week

Once a week: Once a week/2-3 times a months

Not now/special occasions: Less often; only on special occasions/ newer now a days

No: Never had an alcoholic drink

Age 42 Frequency of smoking

(Q: ‘Cohort Member (CM) current smoking status’ with 4 possible answers)

Then it was grouped into 3 categories:

Every day: CM Smokes cigarettes every day

Used to/special occasions: Used to but don’t at all now/ Smokes cigarettes occasionally

No: Never smoked cigarettes

Age 42 Mental well-being - measured by Malaise scale (9-question version) answers Yes/ No

Scoring higher means worse mental well-being; we have dichotomised the score with cut-off point at 4 showing that those who scored 4 and more have worse mental well-being.

*Questions for 9-question version of Malaise scale:*

Whether CM feels tired most of the time

Whether CM often feels miserable and depressed

Whether CM often gets worried about things

Whether CM often gets into a violent rage

Whether CM often suddenly scared for no good reason

Whether CM is easily upset or irritated

Whether CM is constantly keyed up and jittery

Whether every little thing gets on CM's nerves

Whether CM's heart often races like mad

(CM=cohort member)
