# Supplementary material for: Is mid-life social participation associated with cognitive function at age 50? Results from the British National Child Development Study (NCDS)
Source: BMC Psychol. 2016 Dec 2;4:58. doi: 10.1186/s40359-016-0164-x (PMC5134123; doi:10.1186/s40359-016-0164-x)
Supplement: Additional file 3: Table S3. — Sample description bivariates. Description and bivariate association from linear regression between each theory-driven independent variable considered for entry and cognition at age 50. Description and bivariate association from linear regression analyses. [file 40359_2016_164_MOESM3_ESM.docx]

| **Supplementary file Table 3. Description and bivariate association from linear regression between each theory-driven independent variable considered for entry and cognition at age 50** | | | | | | | | | | | | | |
| --- | --- | --- | --- | --- | --- | --- | --- | --- | --- | --- | --- | --- | --- |
|  | | | | | | | N or % | | Unstand B | t | p-value | F-test | 95% CI |
| Standardized score of cognition at age 11 | | | | | | Per unit | 8,448 | | 1.03 | 15.95 | <0.0001 |  | 0.91 to 1.16 |
| (categories not used in analyses; here only for description) | Copying | | | | | Per unit | 8,451 | | 0.32 | 11.37 | <0.0001 |  | 0.27 to 0.38 |
|  | Reading | | | | | Per unit | 8,464 | | 0.87 | 33.00 | <0.0001 |  | 0.82 to 0.92 |
|  | Maths | | | | | Per unit | 8,464 | | 0.84 | 32.66 | <0.0001 |  | 0.79 to 0.89 |
|  | General ability | | | | | Per unit | 8,465 | | 0.89 | 33.51 | <0.0001 |  | 0.83 to 0.94 |
| Gender | | | | | | Male | 49.0 | |  |  |  |  | 0 (ref) |
|  |  |  |  |  |  | Female | 51.0 | | 0.47 | 9.08 | <0.0001 |  | 0.37 to 0.57 |
| **Social network** | | | | | | | | | | | | | |
| Age 33 Household size  (N=7,927) | | | | | 1 | | 1.8 | |  | | |  | 0 (ref) |
|  |  |  |  |  | 2 | | 7.0 | | 0.24 | 1.05 | 0.293 |  | -0.21 to 0.70 |
|  |  |  |  |  | 3 | | 19.0 | | 0.19 | 0.89 | 0.375 |  | -0.23 to 0.61 |
|  |  |  |  |  | 4+ | | 72.2 | | -0.10 | -0.48 | 0.629 |  | -0.51 to 0.31 |
| Age 50 Household size  (N=9,091) | | | | | 1 | | 10.6 | |  | | | <0.0001 | 0 (ref) |
|  |  |  |  |  | 2 | | 31.8 | | 0.13 | 1.42 | 0.154 |  | -0.05 to 0.31 |
|  |  |  |  |  | 3 | | 26.1 | | 0.22 | 2.35 | 0.019 |  | 0.04 to 0.41 |
|  |  |  |  |  | 4+ | | 31.5 | | 0.37 | 4.08 | <0.0001 |  | 0.19 to 0.55 |
| Age 33 Has at least 1 member of family could turn to for advice (N=9,119) | | | | | No/not mentioned | | 23.0 | |  | | |  | 0 (ref) |
|  |  |  |  |  | Yes | | 77.0 | | 0.46 | 7.52 | <0.0001 |  | 0.34 to 0.58 |
| Age 33 Has at least 1 friend/ neighbour/ colleague could turn to for advice (N= 9,119) | | | | | No/not mentioned | | 62.0 | |  | | |  | 0 (ref) |
|  |  |  |  |  | Yes | | 38.0 | | 0.41 | 7.70 | <0.0001 |  | 0.30 to 0.51 |
| Age 42 Has somebody could turn to for advice | | | | | No | | 3.3 | |  | | |  | 0 (ref) |
|  |  |  |  |  | Family member | | 75.0 | | 0.6 | 4.01 | <0.0001 |  | 0.31 to 0.90 |
|  |  |  |  |  | Friend/ colleague/  neighbour | | 22.0 | | 0.64 | 4.03 | <0.0001 |  | 0.33 to 0.95 |
| Age 50 Has someone who listen (N=9,353) | | | | | No one | | 2.5 | |  | | | <0.0001 | 0 (ref) |
|  |  |  |  |  | Somewhat/little | | 22.0 | | 0.55 | 3.13 | 0.002 |  | 0.20 to 0.89 |
|  |  |  |  |  | Yes | | 75.5 | | 0.86 | 5.13 | <0.0001 |  | 0.53 to 1.19 |
| Age 50 Visited by friend/ visiting/phone or mail contact in last 2 weeks  (N= 9,071) | | | | | No | | 6.2 | |  |  |  |  | 0 (ref) |
|  |  |  |  |  | Yes | | 93.8 | | 0.85 | 8.17 | <0.0001 |  | 0.65 to 1.06 |
| Visited by friends | | | | | None | | 37.4 | |  |  |  |  | 0 (ref) |
|  |  |  |  |  | 1x /2x | | 42.8 | | 0.24 | 4.32 | <0.0001 |  | 0.13 to 0.35 |
|  |  |  |  |  | 3x and more | | 19.8 | | 0.19 | 2.79 | 0.005 |  | 0.06 to 0.33 |
| Visiting | | | | | None | | 26.4 | |  |  |  |  | 0 (ref) |
|  |  |  |  |  | 1x /2x | | 44.1 | | 0.51 | 8.35 | <0.0001 |  | 0.39 to 0.63 |
|  |  |  |  |  | 3x and more | | 29.5 | | 0.62 | 9.33 | <0.0001 |  | 0.48 to 0.75 |
| Phone/ mail contact | | | | | None | | 11.2 | |  |  |  |  | 0 (ref) |
|  |  |  |  |  | 1x /2x | | 27.0 | | 0.55 | 6.23 | <0.0001 |  | 0.38 to 0.73 |
|  |  |  |  |  | 3x and more | | 61.8 | | 0.91 | 11.17 | <0.0001 |  | 0.75 to 1.06 |
| Age 33 Married/ Living with partner  (N=7,718) | | Married/ living with partner | | | | | 72.0 | |  |  |  | 0.06 | 0 (ref) |
|  |  | Single/never married | | | | | 17.3 | | 0.01 | 0.07 | 0.94 |  | -0.1 to -0.15 |
|  |  | Separated | | | | | 2.8 | | 0.15 | 0.86 | 0.38 |  | -0.19 to 0.49 |
|  |  | Divorced | | | | | 7.8 | | -0.27 | -2.6 | 0.009 |  | -0.47 to -0.07 |
|  |  | Widowed | | | | | 0.3 | | -0.56 | -1.01 | 0.311 |  | -1.64 to 0.52 |
| Age 42 Married/ Living with partner  (N=8,464) | | Married/ living with partner | | | | | 72.3 | |  |  |  | 0.08 | 0 (ref) |
|  |  | Single/ never married | | | | | 12.3 | | -0.03 | -0.32 | 0.74 |  | -0.19 to 0.14 |
|  |  | Separated | | | | | 2.7 | | -0.09 | -0.56 | 0.58 |  | -0.41 to 0.23 |
|  |  | Divorced | | | | | 12.1 | | -0.22 | -2.74 | 0.006 |  | -0.39 to -0.06 |
|  |  | Widowed | | | | | 0.6 | | 0.23 | 0.67 | 0.5 |  | -0.43 to 0.89 |
| Age 50 Married/ Living with partner  (N=9,114) | | Married/ living with partner | | | | | 68.9 | |  |  |  | 0.008 | 0 (ref) |
|  |  | Single/ never married | | | | | 10.9 | | -0.22 | -2.58 | 0.01 |  | -0.39 to -0.05 |
|  |  | Separated | | | | | 3.2 | | -0.17 | -1.13 | 0.26 |  | -0.46 to 0.12 |
|  |  | Divorced | | | | | 15.6 | | -0.21 | -2.92 | 0.004 |  | -0.35 to -0.07 |
|  |  | Widowed | | | | | 1.4 | | -0.11 | -0.52 | 0.6 |  | -0.53 to 0.31 |
| Age 33 Civic group activities participated in (N= 7,961) | | | | | Per 1 activity + | |  | | 0.56 | 11.10 | <0.0001 |  | 0.46 to 0.66 |
| Age 50 Civic group activities participated in (N=9,117) | | | | | Per 1 activity + | |  | | 0.40 | 13.12 | <0.0001 |  | 0.34 to 0.46 |
| Age 33 Happy relationship (N=5,613) | | | | | Per unit | |  | | 0.02 | 0.75 | 0.453 |  | -0.03 to 0.07 |
| Age 42 Happy relationship (N=6,849) | | | | | Per unit | |  | | 0.01 | 0.96 | 0.336 |  | -0.01 to 0.18 |
| Age 50 Happy relationship (N= 7,608) | | | | | Per unit | |  | | 0.03 | 1.92 | 0.055 |  | -0.001 to 0.07 |
| **Social activities** | | | | | | | | | | | | | |
| Age 50 Watches sport live (N=8,005) | | | | | | Yes | 69.1% | | -0.05 | -0.87 | 0.385 |  | -0.16 to 0.06 |
| Age 50 Goes to cinema (N=8,083) | | | | | | Yes | 52.7% | | -0.68 | -13.05 | <0.0001 |  | -0.78 to -0.58 |
| Age 50 Goes to theatre/concerts (N=8,088) | | | | | | Yes | 44.1% | | -0.78 | -15.12 | <0.0001 |  | -0.89 to -0.69 |
| Age 50 Goes to pub (N=8,133) | | | | | | Yes | 76.4% | | 0.11 | 1.86 | 0.063 |  | -0.010.24 |
| **Takes part in sporting activities and how frequently** | | | | | | | | | | | | | |
| Age 33  (N=7,872) | | | | | | No | 24.9 | |  |  |  | <0.0001 | 0 (ref) |
|  |  |  |  |  |  | 2-3x/month | 6.4 | | 0.43 | 3.67 | <0.0001 |  | 0.20 to 0.66 |
|  |  |  |  |  |  | 1x/week | 21.9 | | 0.52 | 6.63 | <0.0001 |  | 0.37 to 0.67 |
|  |  |  |  |  |  | 2-3/week | 21.3 | | 0.52 | 6.64 | <0.0001 |  | 0.37 to 0.68 |
|  |  |  |  |  |  | 4x-every day | 25.6 | | 0.41 | 5.38 | <0.0001 |  | 0.26 to 0.56 |
| Age 42  (N=8,455) | | | | | | No | 28.0 | |  |  | <0.0001 | <0.0001 | 0 (ref) |
|  |  |  |  |  |  | 2-3x/month | 6.0 | | 0.51 | 4.59 | <0.0001 |  | 0.29 to 0.73 |
|  |  |  |  |  |  | 1x/week | 19.0 | | 0.50 | 6.53 | <0.0001 |  | 0.35 to 0.65 |
|  |  |  |  |  |  | 2-3/week | 21.0 | | 0.62 | 8.35 | <0.0001 |  | 0.47 to 0.76 |
|  |  |  |  |  |  | 4x-every day | 26.0 | | 0.41 | 5.85 | <0.0001 |  | 0.27 to 0.55 |
| Age 50  (N= 9,071) | | | | | | No | 25.1 | |  |  |  | <0.0001 | 0 (ref) |
|  |  |  |  |  |  | 2-3x/month | 6.0 | | 0.66 | 5.67 | <0.0001 |  | 0.43 to 0.88 |
|  |  |  |  |  |  | 1x/week | 21.9 | | 0.62 | 7.65 | <0.0001 |  | 0.46 to 0.78 |
|  |  |  |  |  |  | 2-3/week | 21.0 | | 0.71 | 9.9 | <0.0001 |  | 0.58 to 0.86 |
|  |  |  |  |  |  | 4x-every day | 26.0 | | 0.54 | 8.21 | <0.0001 |  | 0.41 to 0.67 |
| **Health and health behaviour** | | | | | | | | | | | | | |
| Age 33 Self-rated health (N=7,843) | | | | Poor | | | 1.0 | |  |  |  | <0.0001 | 0 (ref) |
|  |  |  |  | Fair | | | 11.0 | | 0.18 | 0.70 | 0.482 |  | -0.32 to 0.68 |
|  |  |  |  | Good | | | 52.0 | | 0.68 | 2.81 | 0.005 |  | 0.21 to 1.16 |
|  |  |  |  | Excellent | | | 36.0 | | 0.93 | 3.81 | <0.0001 |  | 0.45 to 1.41 |
| Age 50 Self-rated health (N=9,070) | | | | Poor | | | 5.7 | |  |  |  | <0.0001 | 0 (ref) |
|  |  |  |  | Fair | | | 12.7 | | 0.50 | 3.93 | <0.0001 |  | 0.25 to 0.74 |
|  |  |  |  | Good | | | 29.2 | | 0.81 | 6.99 | <0.0001 |  | 0.58 to 1.03 |
|  |  |  |  | Very good | | | 33 | | 1.13 | 9.78 | <0.0001 |  | 0.90 to 1.34 |
|  |  |  |  | Excellent | | | 19.4 | | 1.46 | 12.15 | <0.0001 |  | 1.22 to 1.70 |
| Age 33 Frequency of drinking alcohol  (N=7,910) | | | | Never | | | 4.5 | |  |  |  | <0.0001 | 0 (ref) |
|  |  |  |  | Used to/ Occasionally | | | 16.8 | | -0.11 | -0.77 | 0.440 |  | -0.40 to 0.17 |
|  |  |  |  | 1x /week-3x/ month | | | 19.5 | | 0.14 | 1.00 | 0.316 |  | -0.14 to 0.43 |
|  |  |  |  | Daily | | | 59.2 | | 0.24 | 1.82 | 0.069 |  | -0.02 to 0.52 |
| Age 42 Frequency of drinking alcohol  (N=8,459) | | | | Never | | | 1.0 | |  |  |  | <0.0001 | 0 (ref) |
|  |  |  |  | Used to/ Occasionally | | | 16.0 | | 0.08 | 0.34 | 0.737 |  | -0.39 to 0.55 |
|  |  |  |  | 1x /week-3x/ month | | | 30.0 | | 0.3 | 1.29 | 0.197 |  | -0.16 to 0.76 |
|  |  |  |  | Daily/nearly daily | | | 53.0 | | 0.73 | 3.13 | 0.002 |  | 0.27 to 1.19 |
| Age 50 Frequency of drinking alcohol  (N= 9,072) | | | | Never | | | 1.1 | |  |  |  | <0.0001 | 0 (ref) |
|  |  |  |  | Used to/ Occasionally | | | 20.7 | | 0.1 | 0.43 | 0.665 |  | -0.38 to 0.59 |
|  |  |  |  | 1x /week-3x/ month | | | 24.3 | | 0.52 | 2.13 | 0.033 |  | 0.04 to 1.00 |
|  |  |  |  | Daily | | | 53.9 | | 0.89 | 3.67 | <0.0001 |  | 0.41 to 1.36 |
| Age 42 Frequency of smoking (N=8,464) | | | | Never | | | 46.0 | |  |  |  | <0.0001 | 0 (ref) |
|  |  |  |  | Used to/ Occasionally | | | 30.0 | | -0.07 | -1.09 | 0.274 |  | -0.18 to 0.05 |
|  |  |  |  | Daily | | | 24.0 | | -0.58 | -8.93 | <0.0001 |  | -0.71 to -0.45 |
| Age 50 Frequency of smoking  (N= 9,072) | | | | No | | | 46.3 | |  |  |  | <0.0001 | 0 (ref) |
|  |  |  |  | Used to/ Occasionally | | | 34.3 | | -0.10 | -1.88 | 0.06 |  | -0.21 to 0.004 |
|  |  |  |  | Daily | | | 19.4 | | -0.66 | -9.82 | <0.0001 |  | -0.79 to -0.53 |
| Age 33 Body-mass index  (N=7,689) | | | | Underweight | | | 2.4 | |  |  |  | <0.0001 | 0 (ref) |
|  |  |  |  | Normal | | | 54.2 | | 0.12 | 0.65 | 0.517 |  | -0.24 to 0.47 |
|  |  |  |  | Overweight | | | 31.8 | | -0.26 | -1.41 | 0.160 |  | -0.62 to 0.10 |
|  |  |  |  | Obese | | | 11.6 | | -0.21 | -1.09 | 0.277 |  | -0.59 to 0.17 |
| Age 42 Body-mass index (N=7,834) | | | | Underweight | | | 1.1 | |  |  |  | <0.0001 | 0 (ref) |
|  |  |  |  | Normal | | | 46.9 | | -0.34 | -1.32 | 0.188 |  | -0.85 to 0.17 |
|  |  |  |  | Overweight | | | 36.2 | | -0.61 | -2.36 | 0.018 |  | -1.13 to -0.10 |
|  |  |  |  | Obese | | | 15.7 | | -0.78 | -2.94 | 0.003 |  | -1.30 to -0.26 |
| Age 33 Mental well-being  (Malaise score; 9-item version) (N=7,852) | | | | Per unit | | |  | | -0.61 | -5.35 | <0.0001 |  | -0.83 to -0.38 |
|  |  |  |  | Better (0-3) | | | 93.9 | |  |  |  |  | 0 (ref) |
|  |  |  |  | Worse (4+) | | | 6.1 | | -0.61 | -5.21 | <0.0001 |  | -0.84 to -0.38 |
| Age 42 Mental well-being  (Malaise score; 9-item version) (N=8,130) | | | | Per unit | | |  | | -0.08 | -5.14 | <0.0001 |  | -0.12 to -0.05 |
|  |  |  |  | Better (0-3) | | | 89.0 | |  |  |  |  | 0 (ref) |
|  |  |  |  | Worse (4+) | | | 11.0 | | -0.42 |  | <0.0001 |  | -0.62 to -0.31 |
| Age 50 Mental well-being  (Malaise score; 9-item version) (N=8,976) | | | | Per unit | | |  | | -0.57 | -8.07 | <0.0001 |  | -0.71 to -0.43 |
|  |  |  |  | Better (0-3) | | | 85.2 | |  |  |  |  | 0 (ref) |
|  |  |  |  | Worse (4+) | | | 14.8 | | -0.57 | -7.78 | <0.0001 |  | -0.71 to -0.42 |
| Age 33 Often miserable/depressed?  (N=7,875) | | | | | | No |  | |  |  |  |  | 0 (ref) |
|  |  |  |  |  |  | Yes | 11.5 | | -0.39 | -4.41 | <0.0001 |  | -0.56 to -0.21 |
| Age 42 Often miserable/depressed?  (N=8,404) | | | | | | No |  | |  |  |  |  | 0 (ref) |
|  |  |  |  |  |  | Yes | 20.2 | | -0.32 | -4.92 | <0.0001 |  | -0.45 to -0.19 |
| Age 50 Often miserable/depressed?  (N=8,981) | | | | | | No |  | |  |  |  |  | 0 (ref) |
|  |  |  |  |  |  | Yes | 19.1 | | -0.40 | -6.29 | <0.0001 |  | -0.53 to -0.28 |
| Age 33 Epilepsy/fit?  (N=7,876) | | | | | | No |  | |  |  |  |  | 0 (ref) |
|  |  |  |  |  |  | Yes | 1.4 | | -0.84 | -3.24 | 0.001 |  | -1.34 to -0.33 |
| Age 42 Epilepsy/fit?  (N=8,459) | | | | | | No |  | |  |  |  |  | 0 (ref) |
|  |  |  |  |  |  | Yes | 2.2 | | -0.87 | -4.54 | <0.0001 |  | -1.24 to -0.49 |
| Age 50 Epilepsy/fit?  (N=9,091) | | | | | | No |  | |  |  |  |  | 0 (ref) |
|  |  |  |  |  |  | Yes | 0.9 | | -1.06 | -3.75 | <0.0001 |  | -1.61 to -0.51 |
| Age 33 Heart troubles  (N=8,102) | | | | | | No |  | |  |  |  |  | 0 (ref) |
|  |  |  |  |  |  | Yes | 0.2 | | -0.02 | -0.09 | 0.928 |  | -0.41 to 0.37 |
| **Age 44 Biological measurements** | | | | | | | | | | | | | |
| Age 44 Blood pressure  (N= 6,321) | | | | | | Normal | | 85.2 |  |  | |  | 0 (ref) |
|  |  |  |  |  |  | High (>90/140) | | 14.8 | -0.34 | -3.98 | <0.0001 |  | -0.51 to -0.17 |
| Age 44 Waist circumference  (N= 7,369) | | | | | | Normal | | 65.2 |  |  | |  | 0 (ref) |
|  |  |  |  |  |  | Larger  (M>102cm;F>88cm) | | 34.8 | -0.24 | -4.06 | <0.0001 |  | -0.35 to -0.12 |
| **Age 44 Biological markers** | | | | | | | | | **MEAN** | **SD** |  |  |  |
| Cholesterol (N=6,458) | | | | | | Per unit | |  | 5.87 | 1.09 | 0.005 |  | -0.13 to -0.02 |
| Triglycerides (N=6,438) | | | | | | Per unit | |  | 2.04 | 1.55 | <0.0001 |  | -0.13 to -0.05 |
| LDL (N=6,102) | | | | | | Per unit | |  | 3.41 | 0.91 | 0.014 |  | -0.15 to -0.02 |
| HDL (N=6,446) | | | | | | Per unit | |  | 1.57 | 0.40 | <0.0001 |  | 0.31 to 0.61 |
| **Socio-economic background** | | | | | | | | | | | | | |
| Age 0 Father’s/mother’s social class  (N=7,246) | | | Professional | | | | 2.8 | |  |  |  | <0.0001 | 0 (ref) |
|  |  |  | Managerial-technical | | | | 15.9 | | -0.70 | -4.08 | <0.0001 |  | -1.03 to -0.36 |
|  |  |  | Skilled non-manual | | | | 6.4 | | -0.71 | -3.77 | <0.0001 |  | -1.08 to -0.34 |
|  |  |  | Skilled manual | | | | 45.6 | | -1.00 | -6.17 | <0.0001 |  | -1.31 to -0.68 |
|  |  |  | Partly skilled | | | | 15.2 | | -1.27 | -7.35 | <0.0001 |  | -1.60 to -0.93 |
|  |  |  | Unskilled | | | | 14.1 | | -1.47 | -8.40 | <0.0001 |  | -1.81 to -1.12 |
| Age 7 Father’s (male head) social class  (N=8,155) | | | Professional | | | | 5.2 | |  |  |  | <0.0001 | 0 (ref) |
|  |  |  | Managerial-technical | | | | 14.3 | | -0.54 | -4.2 | <0.0001 |  | -0.78 to -0.29 |
|  |  |  | Skilled non-manual | | | | 9.7 | | -0.68 | -4.97 | <0.0001 |  | -0.94 to -0.41 |
|  |  |  | Skilled manual | | | | 44.1 | | -1.23 | -10.65 | <0.0001 |  | -1.46 to -1.01 |
|  |  |  | Partly skilled | | | | 17.4 | | -1.40 | -11.10 | <0.0001 |  | -1.64 to -1.15 |
|  |  |  | Unskilled | | | | 6.4 | | -1.76 | -11.38 | <0.0001 |  | -2.07 to -1.46 |
|  |  |  | No male head | | | | 2.9 | | -1.06 | 5.32 | <0.0001 |  | -1.45 to -0.69 |
| Age 11 Father’s social class  (N= 8,249) | | | Professional | | | | 5.8 | |  |  |  | <0.0001 | 0 (ref) |
|  |  |  | Managerial-technical | | | | 19.2 | | -0.40 | -3.17 | <0.0001 |  | -0.64 to -0.15 |
|  |  |  | Skilled non-manual | | | | 9.5 | | -0.65 | -4.68 | <0.0001 |  | -0.93 to -0.38 |
|  |  |  | Skilled manual | | | | 40.2 | | -1.15 | -9.75 | <0.0001 |  | -1.38 to -0.92 |
|  |  |  | Partly skilled | | | | 15.8 | | -1.35 | -10.46 | <0.0001 |  | -1.60 to -1.09 |
|  |  |  | Unskilled | | | | 5.0 | | -1.66 | -10.25 | <0.0001 |  | -1.98 to -1.34 |
|  |  |  | No male head | | | | 4.5 | | -1.04 | -6.23 | <0.0001 |  | -1.37 to -0.71 |
| Age 7 Accommodation tenure  (N=8,173) | | | Owner occupied | | | | 42.2 | |  |  |  | <0.0001 | 0 (ref) |
|  |  |  | Council rented | | | | 39.8 | | -0.75 | -12.89 | <0.0001 |  | -0.87 to -0.64 |
|  |  |  | Private rented | | | | 12.4 | | -0.60 | -6.99 | <0.0001 |  | -0.77 to -0.43 |
|  |  |  | Rent free | | | | 2.2 | | -0.38 | -2.16 | 0.031 |  | -0.72 to -0.03 |
|  |  |  | Other | | | | 3.3 | | -0.38 | -2.50 | 0.013 |  | -0.67 to -0.08 |
| Age 11 Accommodation tenure  (N=8,342) | | | Owner occupied | | | | 48.0 | |  |  |  | <0.0001 | 0 (ref) |
|  |  |  | Council rented | | | | 40.0 | | -0.75 | -13.11 | <0.0001 |  | -0.86 to -0.64 |
|  |  |  | Private rented | | | | 7.0 | | -0.48 | -4.59 | <0.0001 |  | -0.69 to -0.28 |
|  |  |  | Rent free | | | | 5.0 | | -0.31 | -2.41 | 0.016 |  | -0.56 to -0.06 |
| Age 42 Own social class  (N= 7,297) | | | Professional | | | | 5.0 | |  |  |  | <0.0001 | 0 (ref) |
|  |  |  | Managerial-technical | | | | 39.0 | | -0.48 | -4.0 | <0.0001 |  | -0.72 to -0.24 |
|  |  |  | Skilled non-manual | | | | 22.0 | | -0.91 | -7.27 | <0.0001 |  | -1.16 to -1.41 |
|  |  |  | Skilled manual | | | | 19.0 | | -1.67 | -13.03 | <0.0001 |  | -1.91 to -1.41 |
|  |  |  | Partly skilled | | | | 12.0 | | -1.52 | -11.22 | <0.0001 |  | -1.73 to -1.26 |
|  |  |  | Unskilled | | | | 3.0 | | -2.16 | -11.07 | <0.0001 |  | -2.55 to -1.78 |
| Age 50 Own social class  (N= 9,094) | | | Professional | | | | 5.3 | |  |  |  | <0.0001 | 0 (ref) |
|  |  |  | Managerial-technical | | | | 22.0 | | 0.34 | 2.87 | 0.004 |  | 0.11 to 0.57 |
|  |  |  | Skilled non-manual | | | | 7.8 | | -0.41 | -3.01 | 0.003 |  | -0.68 to -0.14 |
|  |  |  | Skilled manual | | | | 4.2 | | -0.18 | -1.14 | 0.253 |  | -0.49 to 0.13 |
|  |  |  | Partly skilled | | | | 25.9 | | -0.62 | -5.31 | <0.0001 |  | -0.84 to -0.39 |
|  |  |  | Unskilled | | | | 19.3 | | -1.10 | -9.19 | <0.0001 |  | -1.33 to -0.86 |
|  |  |  | Unemployed | | | | 15.6 | | -0.97 | -7.90 | <0.0001 |  | -1.21 to -0.73 |
| Age 50 Highest achieved qualification  (N=9,113) | | | none | | | | 11.0 | |  |  |  | <0.0001 | 0 (ref) |
|  |  |  | CSE or equivalent | | | | 11.0 | | 0.35 | 3.40 | 0.001 |  | 0.15 to 0.55 |
|  |  |  | GCSE or equivalent | | | | 25.0 | | 0.93 | 10.63 | <0.0001 |  | 0.76 to 1.10 |
|  |  |  | AS/A level or equivalent | | | | 17.0 | | 1.31 | 14.05 | <0.0001 |  | 1.13 to 1.49 |
|  |  |  | Degree/teaching diploma/vocational NVQ4 diploma | | | | 31.0 | | 2.03 | 23.83 | <0.0001 |  | 1.86 to 2.20 |
|  |  |  | Higher degree/NVQ5 diploma | | | | 4.0 | | 2.51 | 18.55 | <0.0001 |  | 2.25 to 2.78 |

Due to the mix of categorical and continuous variables frequencies are rounded to 1 decimal place
