# Supplementary material for: Is mid-life social participation associated with cognitive function at age 50? Results from the British National Child Development Study (NCDS)
Source: BMC Psychol. 2016 Dec 2;4:58. doi: 10.1186/s40359-016-0164-x (PMC5134123; doi:10.1186/s40359-016-0164-x)
Supplement: Additional file 4: Table S4. — Change tables cognition. Distribution of categorised cognitive scores. Cognitive score changes between ages 11 and 50. (DOCX 18 kb) [file 40359_2016_164_MOESM4_ESM.docx]

**Additional file 4: Table S4: Distribution of categorised cognitive scores**

1. **Changes in categories of cognition (in %)**

| Age 11 cognitive scores | Age 50 cognitive scores | | |  |
| --- | --- | --- | --- | --- |
|  | Below mean | Mean | Above mean | **Total** |
| Below mean | 47.4 | 35.9 | 16.8 | 28.3 |
| Mean | 31.6 | 41.8 | 26.6 | 35.2 |
| Above mean | 17.7 | 37.7 | 44.7 | 36.5 |
| **Total** | 31.0 | 38.6 | 30.4 |  |

1. **Changes in levels of cognitive ability between age 11 and 50**

|  | N | % |
| --- | --- | --- |
| Deterioration by 2 levels | 523 | 6.4 |
| Deterioration by 1 level | 2,022 | 24.9 |
| The same level | 3,611 | 44.4 |
| Improvement by 1 level | 1,587 | 19.5 |
| Improvement by 2 levels | 386 | 4.8 |
| N | 8,129 | 100.00 |

Cognitive ability was categorised into ‘below mean’, ‘mean’ and ‘above mean’ for both ages.
